# Production of H5N1 Influenza Virus Matrix Protein 2 Ectodomain Protein Bodies in Tobacco Plants and in Insect Cells as a Candidate Universal Influenza Vaccine

**DOI:** 10.3389/fbioe.2015.00197

**Published:** 2015-12-08

**Authors:** Sandiswa Mbewana, Elizabeth Mortimer, Francisco F. P. G. Pêra, Inga Isabel Hitzeroth, Edward P. Rybicki

**Affiliations:** ^1^Biopharming Research Unit, Department of Molecular and Cell Biology, University of Cape Town, Rondebosch, South Africa; ^2^Institute of Infectious Disease and Molecular Medicine, Faculty of Heath Science, University of Cape Town, Cape Town, South Africa

**Keywords:** influenza A virus, M2e, plant expression, insect cell expression, vaccine

## Abstract

The spread of influenza A viruses is partially controlled and prevented by vaccination. The matrix protein 2 ectodomain (M2e) is the most conserved sequence in influenza A viruses, and is therefore a good potential target for a vaccine to protect against multiple virus subtypes. We explored the feasibility of an M2e-based universal influenza A vaccine candidate based on the highly pathogenic avian influenza A virus, H5N1. A synthetic M2e gene was human- and plant-codon optimized and fused in-frame with a sequence encoding the N-terminal proline-rich domain (Zera^®^) of the γ-zein protein of maize. Zera^®^M2e was expressed transiently in *Nicotiana benthamiana* and *Sf*21 baculovirus/insect cell expression systems, and Zera^®^M2e protein bodies (PBs) were successfully produced in both expression systems. The plant-produced Zera^®^M2e PBs were purified and injected into Balb/c mice. Western blot analysis using insect cell-produced Zera^®^M2e PBs and multiple tandem M2e sequences (5xM2e) fused with the avian influenza H5N1 transmembrane and cytosolic tail (5xM2e_tHA) confirmed the presence of M2e-specific antibodies in immunized mice sera. The immunogenicity of the Zera^®^M2e indicates that our plant-produced protein has potential as an inexpensive universal influenza A vaccine.

## Introduction

Influenza A viruses can be highly contagious, causing acute viral respiratory diseases seasonally in the human population (Cox and Subbarao, 1999; Thompson et al., 2003). Currently, vaccination with selected inactivated influenza virus strains is the most effective way of reducing the morbidity and mortality caused by these viruses (Cox and Subbarao, 1999). Influenza A viruses are divided into subtypes by their two surface glycoproteins, the hemagglutinin (HA) and neuraminidase (NA). HA and NA are the primary targets for vaccine development as they elicit neutralizing immune responses (Johansson et al., 1989; Fiore et al., 2009). Unfortunately, due to the high mutation rate of these glycoproteins, influenza vaccines need to be manufactured seasonally in order to be effective against the current circulating strains (Webster et al., 1992). It would therefore be ideal to develop a universal vaccine that is cross-protective against multiple influenza A virus strains as well as against subtypes (Price et al., 2010; Andersson et al., 2012).

There is a type III integral membrane protein (M2) present on the surface of the influenza A virus particle (Lamb et al., 1985). It functions as a pH-activated ion channel (viroporin) and is required for viral infection (Black et al., 1993). It also prevents low pH-induced structural changes in HA during maturation (Sugrue and Hay, 1991), and thus plays a role in viral assembly (Chen et al., 2008; Rossman et al., 2010). M2 (97 amino acids) consists of an N-terminal ectodomain (M2e) (23 amino acids), a lipid bilayer spanning single transmembrane domain (19 amino acids) and a C-terminal cytosolic tail (54 amino acids), and polymerizes into homotetramers in the virion envelope (Pinto and Lamb, 2006). The M2e sequence is highly conserved in all influenza A viruses (Black et al., 1993; Betakova, 2007). It also plays an important role in the incorporation into virions (Park et al., 1998), and it can elicit antibodies that can neutralize virion infectivity (Fiers et al., 2004; Feng et al., 2006). Thus, this domain has the potential to be used as an influenza A virus universal vaccine. However, M2e is covered by the HA and NA proteins in intact virions, and it is therefore unable to react effectively with immune effector cells, making it poorly immunogenic (Lamb et al., 1985; Jegerlehner et al., 2004; Feng et al., 2006). In attempts to enhance its immunogenicity, M2e has been linked to different carrier molecules, such as the TLR5 ligand flagellin (Huleatt et al., 2007; Mardanova et al., 2015), the surface of virus-like particles (VLPs) (Matic et al., 2011), and as a fusion peptide on β-glucuronidase (Firsov et al., 2015) and HA (Stanekova et al., 2011). De Filette et al. (2005) used hepatitis B virus core protein (HBc) as a carrier, and fused M2e to either the C-terminal of HBc or inserted in the immune dominant loop of HBc. Immunization of mice with this HBc-M2e candidate vaccine resulted in 100% protection against lethal challenge (Fiers et al., 2004; De Filette et al., 2005, 2008).

Influenza antigens have been successfully produced using insect cell expression systems. Baculovirus-expressed influenza vaccines can be produced rapidly, which is necessary when taking into account that currently circulating strains need to be assessed annually. The resultant insect cell-expressed product is considered to be both safe and of a high standard (Safdar and Cox, 2007; Cox, 2008). Producing M2e tetramers in baculovirus insect cell expression systems and accumulating them into nanoclusters results in increased humoral and cellular immunogenicity (Wang et al., 2014).

As an alternative approach, numerous researchers have successfully produced influenza antigens in tobacco plants – and in particular HA (D’Aoust et al., 2008; Shoji et al., 2008; Mortimer et al., 2012). Plant expression systems are advantageous due to their ability to carry out post-translational modifications similar to other eukaryotes, and in rapidly producing large quantities of antigen. This system is more economical since plants do not need expensive material for growth and maintenance, and it reduces concerns over human pathogens contaminating vaccine preparations (Nemchinov and Natilla, 2007; Gomez et al., 2009; Rybicki, 2009). Nemchinov and Natilla (2007) developed a candidate plant-based universal influenza vaccine by displaying the M2e epitope on the capsid protein (CP) of cucumber mosaic virus Ixora strain (CMV-Ix) in a potato virus X (PVX)-based vector. The resulting plant-based chimeric CMV capsids reacted specifically to antibodies raised against the synthetic M2e, indicating the potential of this system.

This study forms part of an ongoing initiative to investigate and establish a rapid-response vaccine production platform to deal with future influenza pandemics in South Africa. The highly pathogenic H5N1 influenza A virus, with a mortality rate of up to 60% in humans (http://www.who.int/), was chosen for this purpose. To date, human-to-human transmissions are limited but the likelihood of H5N1 mutating into a strain that facilitates transfer necessitates efficient pandemic vaccination preparedness strategies and awareness (Webster and Govorkova, 2006; Imai et al., 2013; Kaplan and Webby, 2013). To date, potential plant-produced subunit HA vaccines (Mortimer et al., 2012) as well as HA DNA vaccine candidates (Mortimer et al., 2013) have been created as part of this South African initiative.

Fusion of small or soluble proteins to a signal sequence that drives the assembly and sequestration of the protein bodies (PBs) (Torrent et al., 2009) can significantly increase the immunogenicity of the protein. Accordingly, for this study, we investigated the fusion of a consensus M2e sequence to a signal tag (Zera^®^, ERA Biotech) that targets the recombinant protein to form PBs. This tag has previously been shown to dramatically improve yields of non-structural papillomavirus protein (E7SH) in plants as well as to have adjuvant properties (Whitehead et al., 2014). Zera^®^ had adjuvant activity, whether fused to E7SH or simply added to it, which could be highly advantageous in a vaccine candidate. The N-terminal proline-rich domain of maize γ-zein (Zera^®^ tag) is characterized by 4 domains: these are a 19 amino acid signal peptide, a repeat domain containing 8 repeats of the sequence PPPVHL, a Pro-X domain including numerous proline residues as well as a hydrophobic cysteine rich C-terminal domain, and lastly, a sequence that retains it in the endoplasmic reticulum (ER). This allows for the formation of membrane-bound PBs, thereby protecting the recombinant protein from proteolytic degradation inside the host cells, and concentrating and sequestering the recombinant protein. PBs are easily concentrated and partially purified by simple centrifugation, and the polypeptide is generally water soluble in the presence of reducing agents, which greatly facilitates and simplifies recombinant protein purification (Torrent et al., 2009).

For this study, we determined the feasibility of creating an immunogenic M2e candidate vaccine by transiently expressing Zera^®^M2e PBs in tobacco plants and in insect cells. The protein expressed by recombinant baculovirus cells *Sf21* was used as an experimental control reagent. M2e was fused to Zera^®^ to enable protein purification and to increase the immunogenicity of the protein (Torrent et al., 2009; Whitehead et al., 2014). Codon optimization has been widely used to enhance protein expression in heterologous systems (Gouy and Gautier, 1982). The Zera^®^M2e gene was codon optimized for this study such that it either displayed characteristics of abundantly expressed plant genes (*Nicotiana benthamiana* codon optimized) or human genes (human-codon optimized), as we have found it necessary to empirically determine codon preferences in other studies (Maclean et al., 2007). Immunogenicity of the PBs isolated from plants was established by immunization of mice, and analysis of the immune sera for the presence of antibodies against M2e.

## Materials and Methods

### Identification and Synthesis of Zera^®^M2e Peptide

**Figure 1:**
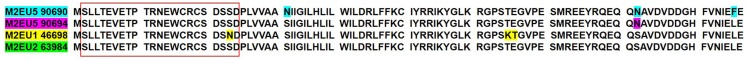
**Avian and human influenza A H5N1 virus M2e sequences retrieved from GenBank and aligned using Clustal X**. EU590690 turkey, EU590684 houbara bustard, EU263984 human, and EU146698 human. The 23 amino acid ectodomain is indicated by the red square. Differences in the amino acid sequence are indicated in different colors.

Multiple avian and human influenza A H5N1 virus M2e sequences were retrieved from GenBank and aligned using Clustal X (Larkin et al., 2007). From these, four sequences were selected (EU590690, EU590684, EU146698, and EU263984) to create a consensus sequence, SLLTEVETPTRNEWECRCSDSSD, which corresponded exactly to the EU263984 sequence [A/human/China/GD02/2006(H5N1)] (Figure 1). To create the Zera^®^M2e sequence, the Zera^®^ sequence (ERA Biotech), including an enterokinase cleavage site (DDDDK) (Whitehead et al., 2014), was synthesized and inserted upstream of the M2e consensus sequence. The Zera^®^M2e nucleotide sequence was both plant- and human-codon optimized, and synthesized by GeneArt (Germany).

### Construction Plant Recombinant Vector

For plant expression, both plant- and human-codon optimized Zera^®^M2e were cloned into the plant expression vector pTRAc (GenBank ID: AY027531) using *Afl*III and *Xho*I restriction enzyme sites (pTRAc-Zera^®^M2e). The pTRAc vector allows protein expression in the cytoplasm (Maclean et al., 2007), with subsequent targeting to the ER by the Zera^®^ sequence. The plasmids were transformed into *E. coli* DH5α and recombinant bacterial colonies were confirmed by PCR using Zera^®^M2e primers (Fw: 5′-ATGCGGGTGCTGCTGGTC-3′ and Rev: 5′-TGGGTGTCTCCACCTCGGTC-3′). The integrity of the plasmids was confirmed by restriction digest mapping with *Xho*I and *Afl*III restriction enzymes as well as sequencing. The pTRAc-Zera^®^M2e plasmids were subsequently transformed into *Agrobacterium tumefaciens GV3101* via electroporation (Maclean et al., 2007).

### Expression and Purification of Zera^®^M2e in *Nicotiana Benthamiana*

*Agrobacterium tumefaciens*-mediated transient expression in *N. benthamiana* plants was performed according to Mortimer et al. (2012). In short, recombinant plant- and human-codon optimized pTRAc-Zera^®^M2e plasmids were vacuum infiltrated into 6-week-old plants, with co-infiltration of *A. tumefaciens LBA4404* (pBIN-NSs) containing the NSs gene silencing suppressor of tomato-spotted wilt virus (TSWV) (Marcel Prins, Laboratory of Virology, Wageningen, The Netherlands); this enhances gene expression by suppressing post-translational gene silencing (Takeda et al., 2002).

Infiltrated plant tissue was harvested 8 days post infiltration (dpi), followed by grinding in liquid nitrogen with a mortar and pestle, after which the extract was homogenized in the Zera^®^ extraction buffer [100 mM Tris (pH 8), 0.5M NaCl, 50 mM MgCl_2_, and 10 mM EDTA]. The homogenate was filtered through two layers of Miracloth (Merck) and purified by ultracentrifugation (Beckman SW32Ti rotor) at 21,600 × *g* for 2 h through a 60% sucrose cushion.

Protein expression was assessed by western blot analysis, with proteins resolved on 15% SDS-PAGE gels. The primary antibody, rabbit anti-Zera^®^ polyclonal antibody (provided by ERA Biotech, Spain), was used at a dilution of 1:7000 together with a secondary goat anti-rabbit antibody (Sigma, Steinheim, Germany) at 1:7000 dilution. Nitro blue tetrazolium chloride/5-bromo-4 chloro-3-indolyl phosphate (NBT/BCIP) phosphate substrate (KPL, Gaithersburg, MD, USA) was used for detection. Plant-produced Zera^®^M2e was quantified by comparing band intensities of the Zera^®^M2e to known bovine serum albumin (BSA) concentrations by gel densitometry (Gene Genius Bioediting system, Syngene).

### Construction and Expression of Zera^®^M2e in Insect Cells

For insect cell expression, plant- and human-codon optimized Zera^®^M2e was cloned into the pFastBac Dual vector (InVitrogen, Carlsbad, CA, USA) between the polyhedrin (PPH) promoter and Tn7L terminator using *Eco*RI and *Pst*I restriction sites, resulting in pFastBac-Zera^®^M2e. Recombinant plasmids screened by PCR with pFastBac primers (Fw: 5′-GATGGTGGGACGGTATGAATAATCC-3′ and Rev: 5′-GGTATTGTCTCCTTCCGTGTTTGA-3′). The integrity of the plasmids was confirmed by plasmid mapping with *Eco*RI and *Pst*I restriction enzymes and sequencing. Recombinant bacmid DNA was obtained by transposition of pFastBac-Zera^®^M2e into *E. coli* DH10Bac according to the manufactures instructions (InVitrogen, Carlsbad, CA, USA).

Recombinant baculoviruses (rBV) *Sf 21* cells containing plant- and human-codon optimized Zera^®^M2e were generated, and plaque assays to determine rBV titers were performed according to the Bac-to-Bac© baculovirus expression system manufacturer’s protocols (InVitrogen, Carlsbad, CA, USA). TC Plates were stained with 1 g/ml neutral red solution (Sigma, Steinheim, Germany) to visualize individual plaques. Protein expression and purification analysis are as described for the plant-produced proteins.

### Animal Trials and Serum Analysis

Only the plant-produced Zera^®^M2e PB yields were judged to be sufficient for animal trials. Accordingly, 20 female Balb/c mice (7 weeks old) were divided into two groups: (a) plant-produced Zera^®^M2e PB and (b) PBS negative control group. A dose of 4.5 μg Zera^®^M2e PB was administered intramuscularly (I.M.) to mice, into each anterior *tibialis* muscle. Four doses were administered at 2-week intervals on days 0, 14, 28, and 31. Pre-vaccination serum was collected 3 days prior to vaccination. Following vaccination, sera were collected before each boost (on days 14, 28, and 31), and stored at −20°C until for further analysis. Eleven days after the final dose, animals were euthanized (day 42). The animal experiments were approved by the University of Cape Town’s (UCT) Animal Ethics Committee (HSFAEC 009/001).

Western blots were performed to determine the presence of Zera^®^M2e-specific antibodies in the mouse sera. Our insect cell- and plant-produced Zera^®^M2e PB samples were resolved separately on 15% SDS-PAGE gels, followed by transfer onto a nylon membrane (Armersham, Bioscience, UK) by semi-dry blotting (BioRad Hercules, CA, USA). The membranes were cut into individual strips and were incubated in 1:5000 dilutions of serum from mice injected with pTRAc-Zera^®^M2e PB and PBS, respectively. The secondary goat anti-mouse antibody (Sigma, Steinheim, Germany) was used at 1:7000 dilutions. As a positive control, the commercial rabbit polyclonal anti-M2 antibody (ab65086) (Abcam, Cambridge, UK) was used at a 1:5000 dilution followed by the secondary goat anti-rabbit antibody (Sigma), at a 1: 7000 dilution.

To assess if the sera did not only bind the Zera^®^ but also the M2e, western blots were performed with a construct encoding multiple M2e (5xM2e) fused with the avian influenza H5N1 (A/Vietnam/1194/2004 H5N1) transmembrane and cytosolic tail (5xM2e_tHA). Crude plant extract containing the 5xM2e_tHA protein was resolved on 12% SDS-PAGE gels, and the protein was probed on blots with a 1:100 sera dilution. As a positive control, 5xM2e_tHA was probed with 1:5000 anti-M2 monoclonal antibody (14C2). Alkaline phosphate-conjugated goat anti-mouse IgG was used as a secondary antibody at a 1:10,000 dilution.

## Results

### Expression of Recombinant Protein in *N. benthamiana*

**Figure 2:**
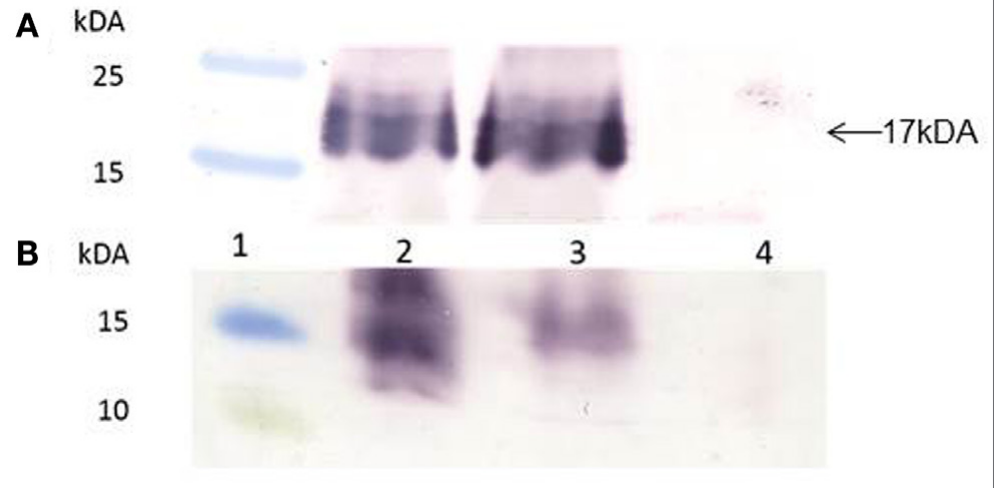
**Western blots of human- and plant-codon optimized recombinant Zera^®^M2e protein bodies (PBs)**. Protein expression was detected with anti-Zera^®^ antibody and goat anti-rabbit antibody. **(A)** Transient expression in *N. benthamiana*. Plants were harvested 8 days post infiltration. Lane 1 contained PageRuler™ Prestained protein ladder (Fermentas), lane 2 contained the Zera^®^M2e plant-codon optimized protein, lane 3 contained the Zera^®^M2e human-codon optimized, and lane 4 contained non-infiltrated control plant. **(B)** Expression of recombinant baculovirus in *Sf*21 insect cells, 72 h post infection (hpi), using the Bac-to-Bac^©^ baculovirus expression system. Lane 1 contained the PageRuler™ Prestained protein ladder (Fermentas), lane 2 contained the recombinant human-codon-optimized Zera^®^M2e PB, lane 3 is the recombinant plant-codon-optimized Zera^®^M2e, and lane 4 contained the negative cell lysate transfection control.

Gene-codon optimization has been shown to significantly enhance gene expression in plants, and especially if the genes have a high GC content. The Zera^®^M2e gene was plant- and human-codon optimized and successfully cloned into plant expression vector pTRAc. Protein expression analysis revealed that both plant- and human-codon optimized Zera^®^M2e PB were successfully expressed in *N. benthamiana* 8 dpi. In western blots, an expected band of 17 kDa corresponding to the M2e epitope fused with Zera^®^ tag was observed. There were no differences in the expression levels between the plant- and human-codon optimized Zera^®^M2e, indicating that codon optimization did not influence gene translation and expression in the plant expression system (Figure 2A).

### Expression of Recombinant Protein in Insect Cells

For insect cell expression, plant- and human-codon optimized Zera^®^M2e were successfully cloned into pFastBac Dual vector under the control of the pH10 promoter and expressed in *Sf*21 insect cells 72 h post infection (hpi) (Figure 2B). The plant-codon optimized gene expression was weak as assessed by western blot compared to the human-codon optimized gene. In this case, human-codon usage was more favorable for the insect cell expression system.

### Purification of Recombinant Protein

**Figure 3:**
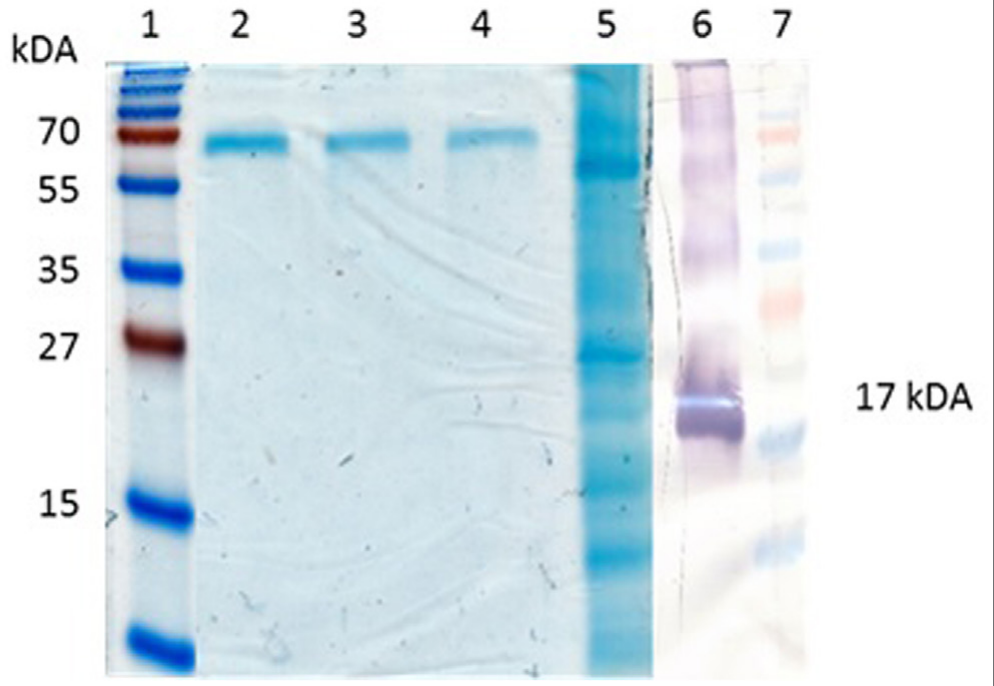
**Quantification of Zera^®^M2e produced in plants**. Bovine serum albumin (BSA) was used as a standard. Lane 1 and 7 contained PageRuler™ Prestained protein ladder (Fermentas), lane 2 contained 6.26 mg/ml, lane 3 contained 3.13 mg/ml, lane 4 contained 1.56 mg/ml, and lane 5 contained partially purified Zera^®^M2e plant-codon optimized protein. While lane 6 is a western blot containing the partially purified Zera^®^M2e plant-codon optimized protein detected with anti-Zera^®^antibody.

The PBs can be easily purified by centrifugation through a sucrose cushion (Torrent et al., 2009). Ultracentrifugal concentration through a sucrose cushion showed that the Zera^®^M2e PBs formed insoluble pellets. The pelleted insect cell-produced Zera^®^M2e was detectable on western blots but was undetectable on Coomassie-stained SDS-PAGE gels, indicating low protein concentration. The concentrated and partially purified plant-produced Zera^®^M2e PB was visible on Coomassie-stained SDS-PAGE gels and was quantified by comparing it to known BSA concentrations run on stained gels (Figure 3). Bands corresponding to the monomeric (17 kDa), dimeric (34 kDa), and tetrameric (51 kDa) forms of the Zera^®^M2e PB were observed (Figure 3). Only the band corresponding to the putative monomeric form was used for quantitation, to give estimated expression levels ranging from 125 to 205 mg Zera^®^M2e PB/kg fresh weight (FW) as measured by densitometry. The higher yielding plant-produced Zera^®^M2e PB was used for animal trials.

### Animal Serum Analysis

Ten mice were immunized with the plant-produced Zera^®^M2e PB. No clinical manifestation was observed after the injection of the PBs in any of the mice. After the fourth immunization, the sera were analyzed for the presence of Zera^®^M2e-specific antibodies. Western blots indicated that the immune sera successfully reacted with the plant-produced Zera^®^M2e PB at a dilution of 1:5000, indicating a high Zera^®^M2e-specific antibody titer. Sera from PBS-inoculated control mice did not bind Zera^®^M2e PB (Figure S1 in Supplementary Material).

**Figure 4:**
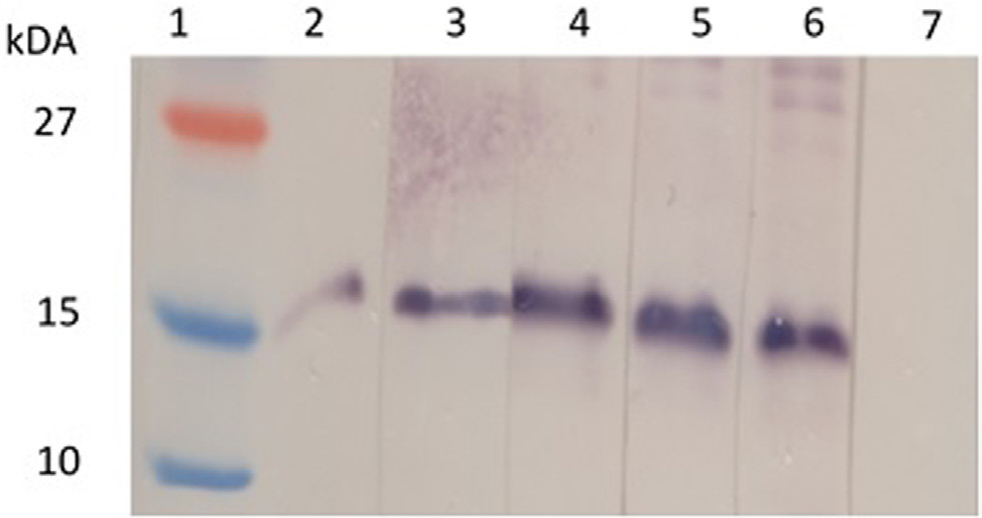
**Detection of Zera^®^M2e-specific antibodies in plant-produced Zera^®^M2e immunized mice sera**. Insect cell-produced Zera^®^M2e protein bodies (PBs) were loaded in each lane, and then the membrane was cut into strips and probed with individual mouse serum. Lane 1 contains PageRuler™ Prestained protein ladder (Fermentas), lane 2 contains the positive control, i.e., Zera^®^M2e PB detected with a commercial M2 primary antibody (1:5000) (ab65086, Abcam, Cambridge, UK). Lane 3–6 were detected with mice sera from mice immunized with plant-produced Zera^®^M2e PB (1:5000) and Lane 7 contains negative control sera: mice immunized with PBS.

When the mouse sera were tested with our plant-produced Zera^®^M2e PB, a high level of background was observed on the western blots even when the sera were diluted 1:40,000, which made it difficult to identify the expected band sizes (Figure S1 in Supplementary Material). This is because our candidate vaccine was produced in plants, and therefore the sera also reacted with high specificity to plant proteins contaminating the preparations. In an attempt to lower the background for more accurate results, the sera were then analyzed using our insect cell-produced Zera^®^M2e PB (Figure 4). The immune sera reacted far more specifically, with a distinct band corresponding to the monomeric Zera^®^M2e PB.

**Figure 5:**
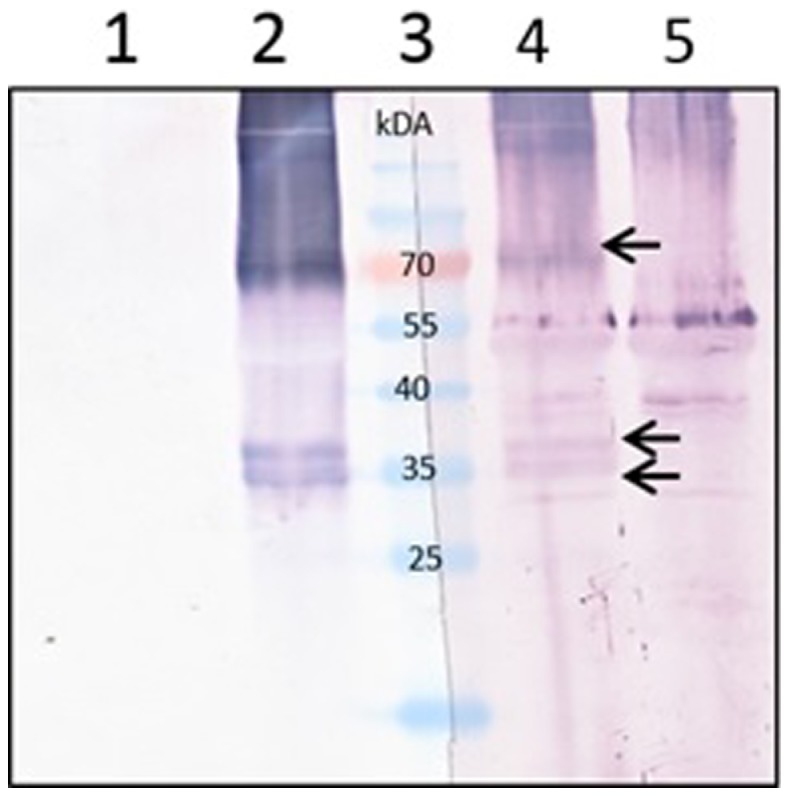
**Confirmation of specificity of the Zera^®^M2e mice sera against the fused M2e plant-produced protein**. Equal volume of plant-produced 5xM2e_tHA proteins were loaded in each lane. The membranes were cut in the middle and each half was probed either with the commercial M2 antibody or mice serum from mice immunized with plant-produced Zera^®^M2e PB. Lanes 1 and 5 contain *Agrobacterium*-infiltrated crude plant extract (negative control), Lanes 2 and 4 contain the 5xM2e_tHA plant crude extract, and Lane 3 contains PageRuler™ Prestained protein ladder (Fermentas). Lanes 1 and 2 were detected with commercial M2 (14C2) primary antibody (1:5000). Lanes 4 and 5 were detected with mice sera from mice immunized with plant-produced Zera^®^M2e PB (1:100).

To be certain whether the mouse sera produced reacted specifically with the M2e peptide, the sera were tested by western blot against the plant-produced 5xM2e_tHA, which contains no Zera^®^ sequence (Figure 5). The serum strongly reacted with the 35-kDa multiple protein dimer and a 70-kDa trimeric form, affirming that indeed the mouse sera did not only have antibodies against Zera^®^ peptide but also against the M2e sequence.

## Discussion

In this study, we attempted to overcome the limitations of the current influenza vaccines with regards to antigenic shift and drift associated with HA and NA (Webster et al., 1992), by focusing on the influenza A virus M2e peptide as a universal vaccine candidate, due to its high degree of conservation since the emergence of the highly virulent Spanish flu pandemic strain of 1918 (Fiers et al., 2004). Generally, M2e vaccine candidates are produced either as chemically conjugated molecules or by genetic fusion to a variety of carrier proteins, such as virion- or VLP-forming proteins (Mozdzanowska et al., 2003; Ionescu et al., 2006; Tompkins et al., 2007; Denis et al., 2008; Matic et al., 2011; Stanekova et al., 2011; Ravin et al., 2012). In our study, we fused the M2e with a self-aggregating signal tag, Zera^®^. The Zera^®^ fusion tag has the ability to segregate the protein from the plant secretory pathway into membrane-delimited organelles in the ER: the retention and the self-assembly of γ-zein lead to PB formation, stabilizing the protein inside vesicles formed by invagination of the ER lumen (Mainieri et al., 2004; Torrent et al., 2009). This is known to facilitate protein purification, as shown by the result of ultracentrifugation of the plant-produced Zera^®^M2e PBs through a 60% sucrose cushion: PBs were concentrated in the pellet fraction, with very little soluble protein present.

As the 2009 H1N1 “swine flu” pandemic illustrated, South Africa will have to rely on developed countries for vaccine supplies during an outbreak (Mortimer et al., 2012, 2013). We are therefore systematically investigating the feasibility of establishing rapid-response platforms to produce influenza virus vaccine candidates in South Africa, by implementing novel strategies that are both cost-effective and can be more readily up-scaled than traditional egg-based vaccines (Mortimer et al., 2012).

In the present work, we were able to express Zera^®^M2e in both plants and insect cells. The protein was expressed as monomers, dimers, and tetramers. This is in line with previous work, where M2e was expressed in baculovirus expression system (Holsinger and Lamb, 1991; Sugrue and Hay, 1991). In plants, we achieved a yield of 125–205 mg/kg FW for the Zera^®^M2e. These are generally higher yields than those that have been achieved by other researchers. Nemchinov and Natilla (2007) previously reported yields of avian influenza A CMV-M2e fusion protein expression in *N. benthamiana* of 6–8 mg/kg leaf tissue. Matic et al. (2011) expressed influenza M2e epitopes on chimeric HPV VLPs in plants and obtained 78–120 mg/kg plant material. Most recently, Firsov et al. (2015) expressed M2e fused with β-glucuronidase in transgenic duckweed, and obtained yields from 90 to 970 mg/kg plant FW.

When the serum from mice vaccinated with plant-produced Zera^®^M2e PB was used to detect the same protein produced in insect cells on western blots, the serum specifically detected only the Zera^®^M2e protein (Figure 4), indicating that the plant-produced antigen was immunogenic. Use of the plant-produced protein, however, showed that the sera also reacted with other plant proteins that co-purified with the Zera^®^M2e PBs. Zera^®^ fusions are known to form large polymers that are resistant to degradation (Torrent et al., 2009). The contaminating proteins could include chloroplastic, ribosomal, cytoplasmic, cytoskeleton, and mitochondrial plant proteins (Joseph et al., 2012). It is clear that using a different expression system for antibody detection from that which was used for the antigen production, allowed for more efficient and clear detection of the protein. Future work will include ultracentrifugation on a sucrose density step gradient (Whitehead et al., 2014) to remove unwanted plant protein: this should then lead to more specific immune responses only to the Zera^®^M2e PB, with reduced reaction against other plant proteins. Testing the Zera^®^M2e antigen for protection against multiple strains of influenza would be advantageous but was not possible in this investigation.

While we successfully produced Zera^®^M2e PB in insect cells in this work, and this was valuable as a reagent, our plant-produced Zera^®^M2e PB had by far the highest yield, with the different soluble forms (monomeric, dimeric, trimeric, and tetrameric) probably contributing significantly to the immunogenicity of the candidate vaccine. Bands corresponding to the monomeric, dimeric, and tetrameric forms have also been detected in previous M2e studies (Holsinger and Lamb, 1991; Sugrue and Hay, 1991).

The production of M2e as a fusion product in plants is not new; Nemchinov and Natilla (2007) expressed M2e in *N. benthamiana* via a plant viral vector as an internal fusion in the CP of Cucumber mosaic virus; Ravin et al. (2012) used a similar vector to express M2e fused to the HBc and showed protection against lethal challenge in mice. Matic et al. (2011) used the pEAQ-*HT* vector to transiently express M2e and a shortened version (M2e_2–9_) as fusions to HPV-16 L1 protein; the longer peptide was presented on capsomers and VLPs, and reacted with anti-M2e antibodies. Petukhova et al. (2013) produced recombinant tobacco mosaic virus particles presenting M2e on their surfaces and showed these were highly immunogenic and protective.

However, our use of the Zera^®^ peptide is novel – moreover, the high yield and the immunogenicity of the product, coupled with what is almost certainly a far easier purification protocol than for any of the fusions detailed above, make it a valuable addition to our rapid-response armory against pandemic influenza. Our work is therefore further proof that plants are a viable vehicle for high-level expression of a peptide vaccine known to elicit broad-spectrum protection against influenza A viruses.

To conclude, we successfully expressed Zera^®^M2e PB in both insect cell and plant expression systems. Our plant-produced Zera^®^M2e elicited M2e-specific antibodies in mice, which indicates that it has potential as a candidate universal influenza vaccine. Future work will look at determining the efficacy of these antibodies and their potential of broad-spectrum protection against influenza strains.

## Author Contributions

SM created the expression constructs, carried out transient expression experiments, and drafted the manuscript; LM carried out insect cell expression, supervised the work, and participated in drafting of the manuscript, FP created and expressed 5xM2e-tHA in plants; IH designed, coordinated, and supervised the study and participated in drafting of the manuscript; ER initiated study and participated in drafting of manuscript.

## Conflict of Interest Statement

The authors declare that the research was conducted in the absence of any commercial or financial relationships that could be construed as a potential conflict of interest.
