# Supplementary material for: Production of H5N1 Influenza Virus Matrix Protein 2 Ectodomain Protein Bodies in Tobacco Plants and in Insect Cells as a Candidate Universal Influenza Vaccine
Source: Front Bioeng Biotechnol. 2015 Dec 8;3:197. doi: 10.3389/fbioe.2015.00197 (PMC4672040; doi:10.3389/fbioe.2015.00197)

Supplementary Figure 1: Detection of Zera®M2e-specific antibodies in plant-produced Zera®M2e immunised mice sera. Plant produced Zera®M2e protein bodies (PB) were loaded in each lane and then the membrane was cut into strips and probed with different dilutions of mouse serum. Lane 1, contains PageRuler™ Prestained protein ladder (Fermentas), lane 2, contains the positive control, i.e. Zera®M2e PB detected with a commercial M2 primary antibody (1:5000) (ab65086, Abcam, Cambridge, UK). Lane 3 – 6, were detected with mice sera at 1: 5 000, 1:10 000; 1:20 000; 1:40 000 dilutions from mice immunized with plant-produced ZeraM2e PB and Lane 7 contains negative control sera: mice immunised with PBS.

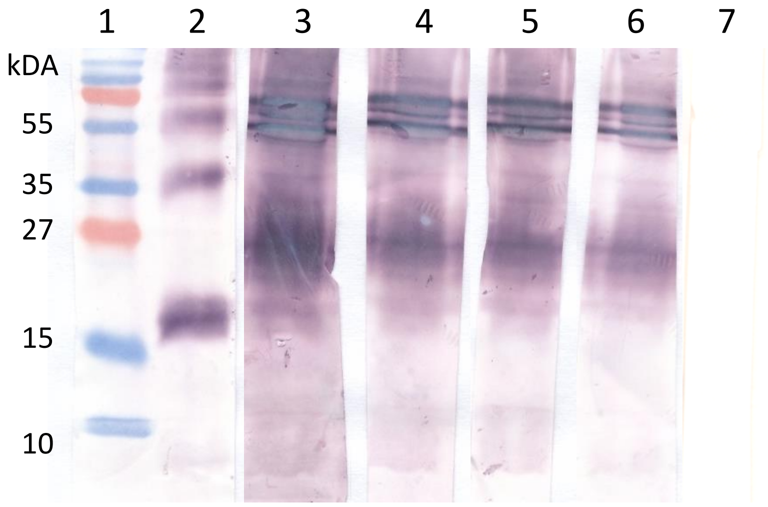

Supplement: Supplementary file 1 [file Image_1.PDF]
